# Intraoperative blood pressure strategies and neurocognitive outcomes: A systematic review

**DOI:** 10.1016/j.jatmed.2026.06.002

**Published:** 2026-06-26

**Authors:** Paula Henríquez Miranda, Daniela Peña Pérez, Juan Pablo Maestre Aguancha, Mariela Alejandra Cabas Ramos, Juan Sebastián Cuellar Cadena, Francisco Javier Gómez Ballesta, Juan Pablo Alzate Granados

**Affiliations:** a Medicine Program, Faculty of Health Sciences, Universidad Simón Bolívar, Barranquilla 080002, Colombia; b Medicine Program, Faculty of Health Sciences, Universidad Libre, Barranquilla 080002, Colombia; c School of Medicine, Fundación Universitaria Sanitas, Bogotá 111711, Colombia; d Medicine Program, Faculty of Health Sciences, Universidad Cooperativa de Colombia, Barranquilla 080002, Colombia; e School of Medicine and Health Sciences, Universidad del Rosario, Bogotá 111711, Colombia; f Faculty of Medicine, Universidad Autónoma de Bucaramanga, Bucaramanga 680003, Colombia; g Faculty of Medicine, Universidad Nacional de Colombia, Bogotá 111711, Colombia

**Keywords:** Intraoperative Care, Blood Pressure, Hypotension, Controlled, Delirium, Neurocognitive Disorders

## Abstract

**Background:**

Intraoperative blood pressure instability during anesthesia may compromise cerebral perfusion and has been associated with postoperative delirium and perioperative neurocognitive disorders. However, randomized evidence remains heterogeneous regarding whether targeted intraoperative blood pressure management strategies reduce postoperative neurocognitive complications compared with standard care.

**Methods:**

We conducted a systematic review of randomized controlled trials evaluating intraoperative blood pressure, perfusion-related, ventilatory, or anesthetic strategies in adult surgical patients. The review was registered in PROSPERO and reported according to PRISMA 2020. Searches were performed in PubMed, EMBASE, and LILACS, with no language or publication-status restrictions. Study selection, data extraction, and risk-of-bias assessment were performed independently by two reviewers. Risk of bias was evaluated using the Cochrane Risk of Bias 2 tool. Because of clinical and methodological heterogeneity in interventions, populations, neurocognitive definitions, and follow-up timing, findings were synthesized narratively without meta-analysis.

**Results:**

Thirteen randomized controlled trials were included. Protocolized interventions, including prophylactic vasopressor infusion, individualized blood pressure targets, ventilatory adjustment, and regional or combined anesthetic techniques, were generally associated with improved hemodynamic stability and selected early perioperative benefits. Individualized blood pressure management reduced altered consciousness from 15.9% to 5.4% in one trial, while prophylactic norepinephrine reduced intraoperative hypotension from 74% to 15% in another. High normocapnia markedly reduced cerebral desaturation events from 55.6% to 8.8%. Trials evaluating sedative or anesthetic regimens, including esketamine, remimazolam, dexmedetomidine, desflurane, sevoflurane, and propofol-based strategies, suggested possible benefits on hemodynamic stability, analgesic requirements, and early recovery markers. However, evidence for sustained reduction in postoperative delirium or long-term perioperative neurocognitive disorders remained limited and inconsistent.

**Conclusions:**

Protocolized intraoperative blood pressure and perfusion-related strategies may improve hemodynamic stability and reduce selected early perioperative complications. Nevertheless, current randomized evidence is insufficient to conclude that these strategies consistently prevent postoperative delirium or long-term perioperative neurocognitive disorders. Future trials should use standardized neurocognitive definitions, validated delirium instruments, longer follow-up, and complete reporting of absolute event rates and effect estimates.

## Introduction

Intraoperative blood pressure (IBP) is a critical determinant of cerebral perfusion during general anesthesia. Cerebrovascular autoregulation maintains relatively constant cerebral blood flow within a range of perfusion pressures, but this margin may shift or narrow in older adults and in patients with chronic hypertension, cerebrovascular disease, or frailty.^1^ In this context, anesthetic interventions that deliberately modify IBP such as controlled hypotension to reduce bleeding or normotensive maintenance to preserve perfusion could directly influence postoperative neurocognitive dysfunction (PND), which includes delirium and early or persistent cognitive decline.^2^, ^3^ Moreover, anesthetic depth, anemia, hemodilution, and the use of vasopressors or vasodilators modulate the relationship between IBP and neurological outcomes, emphasizing the need to evaluate specific hemodynamic control strategies under general anesthesia.^4^, ^5^

Despite physiological plausibility, clinical findings are heterogeneous. Some clinical trials suggest that preventing deep or prolonged hypotensive episodes may reduce delirium; others find no relevant differences compared to conventional strategies.^6^ Variability in definitions, thresholds and duration of hypotension or hypertension, protocols, cardiac and non-cardiac surgical populations, and PND measurement including delirium instruments and neuropsychological test batteries limits comparability and leaves uncertainty about which IBP management strategy offers the best balance between cerebral safety and bleeding control during surgery under general anesthesia.^7^, ^8^

We hypothesized that targeted intraoperative blood pressure management, such as controlled hypotension with explicit targets and close monitoring or normotensive maintenance within predefined limits, may reduce postoperative delirium and perioperative neurocognitive disorders compared with conventional care or approaches without strict blood pressure control. Accordingly, this systematic review aimed to determine, in randomized controlled trials of adult surgical patients receiving anesthesia, whether these strategies are associated with lower rates of delirium and early (≤30 days) or later (>30 days) postoperative cognitive decline, and to explore potential effect modifiers including age, surgical setting, comorbidities, anesthetic exposure, and vasoactive drug choice. Primary outcomes were postoperative delirium, assessed with validated instruments, and perioperative neurocognitive outcomes, measured using standardized cognitive tests as reported by each trial. Secondary outcomes included major perioperative complications, length of stay, reinterventions, and hemodynamic and perfusion related findings such as off target hypotensive or hypertensive episodes. Therefore, we conducted a systematic review to evaluate whether intraoperative blood pressure management strategies are associated with postoperative delirium and perioperative neurocognitive disorders, and to summarize related perioperative secondary outcomes and intermediate hemodynamic findings.

## Methods

### Protocol and reporting framework

This systematic review was registered in PROSPERO under registration number CRD4201265588. The review was conducted and reported in accordance with the PRISMA 2020 statement. The review question focused on randomized controlled trials evaluating whether intraoperative blood pressure management, perfusion-related, ventilatory, or anesthetic strategies in adult surgical patients were associated with postoperative delirium, perioperative neurocognitive disorders, or related early neurological and perioperative outcomes.

### Eligibility criteria

Eligible studies were randomized controlled trials involving adult patients aged 18 years or older undergoing surgical procedures under general, regional, or combined anesthesia. Trials were considered eligible when they evaluated an intraoperative strategy intended to modify blood pressure, systemic or cerebral perfusion, ventilation, hemodynamic stability, or anesthetic exposure in comparison with usual care, placebo, or an alternative intraoperative management strategy. Eligible interventions included absolute or individualized mean arterial pressure targets, prophylactic or rescue vasopressor protocols, hemodynamic goal-directed approaches, cerebral or systemic perfusion-guided strategies, ventilation strategies affecting cerebral oxygenation, and anesthetic or sedative regimens with potential hemodynamic or neurological effects.

The primary outcomes were postoperative delirium assessed using validated instruments and perioperative neurocognitive disorders or postoperative cognitive dysfunction assessed using standardized cognitive criteria, neuropsychological tests, or validated cognitive scales as reported by the original trials. Secondary outcomes included objective cognitive test changes, altered consciousness, awakening or recovery scores, cerebral desaturation events, cerebrovascular events, myocardial infarction, acute kidney injury, mortality, intensive care unit or hospital length of stay, readmission, and adverse events attributable to the intervention. Intermediate hemodynamic and perfusion-related outcomes included hypotension burden, mean arterial pressure stability, vasopressor requirements, cerebral oxygenation, and time or area under predefined hypotension thresholds.

Non-randomized studies, quasi-experimental studies, case series, observational studies, in vitro studies, pediatric studies, and studies focused exclusively on non-surgical critical care were excluded. Crossover trials were excluded when the evaluated outcome involved acute safety, neurocognitive recovery, or a clinically relevant possibility of carryover effects. Multiple reports of the same trial were linked before inclusion decisions to avoid duplication.

### Operational definitions of neurocognitive outcomes

Postoperative delirium was defined as an acute and fluctuating disturbance in attention, awareness, or cognition diagnosed using validated delirium assessment instruments, including the Confusion Assessment Method, Confusion Assessment Method for the Intensive Care Unit, 3D-CAM, CAM-CR, or equivalent tools, as reported by each trial. Delirium severity scores were extracted separately from delirium incidence when available.

Perioperative neurocognitive disorders were used as an umbrella term for postoperative cognitive outcomes assessed beyond the immediate emergence period, including postoperative cognitive dysfunction or cognitive decline according to the terminology, diagnostic criteria, instruments, and assessment timing used in each original trial. When studies used the Mini-Mental State Examination, Montreal Cognitive Assessment, Short Orientation–Memory–Concentration Test, neuropsychological batteries, or other cognitive scales, the specific instrument, cut-off, direction of change, and time point were extracted.

Altered consciousness, early awakening scores, Glasgow Coma Scale, Aldrete score, cerebral desaturation events, early Mini-Mental State Examination changes, and recovery-quality scores were not considered equivalent to long-term perioperative neurocognitive disorders unless the original study explicitly defined them as such using validated neurocognitive criteria. These outcomes were therefore analyzed as early neurological recovery markers, intermediate cerebral perfusion indicators, or secondary outcomes rather than definitive evidence of sustained postoperative neurocognitive dysfunction.

### Search strategy and study identification

Searches were performed in PubMed, EMBASE, and LILACS. No language or publication-status restrictions were applied. The absence of language restrictions was intended to reduce selection bias related to non-English publications. Search strategies combined terms related to surgical patients, intraoperative blood pressure, hypotension, mean arterial pressure, hemodynamic management, perfusion, anesthesia, delirium, postoperative cognitive dysfunction, and perioperative neurocognitive disorders, together with sensitive filters for randomized controlled trials. Full search strategies for all databases are provided in the Supplementary Material.

Reference lists of included studies and relevant reviews were screened to identify additional eligible reports. Conference proceedings and trial records were also considered when available. Authors or investigators were contacted when additional information was required. Before resubmission, an updated search was performed to verify whether relevant high-quality recent randomized trials or seminal studies had been omitted. Recent evidence identified during this update consisted mainly of reviews, meta-analyses, observational studies, or ongoing trial records, and no additional randomized controlled trial meeting the eligibility criteria was incorporated into the final synthesis.

### Study selection and data extraction

Retrieved records were managed using Zotero for deduplication. Two reviewers independently screened titles and abstracts and subsequently assessed potentially eligible full-text reports. Disagreements were resolved by consensus and, when necessary, by a third reviewer. Data extraction was performed independently using a standardized and piloted extraction form, and extracted data were cross-checked for completeness and consistency.

Extracted variables included study design, country, year of publication, sample size randomized and analyzed per outcome, age, sex, American Society of Anesthesiologists physical status, baseline hypertension, cardiovascular or cerebrovascular comorbidities, baseline cognitive status when reported, surgical specialty, type of anesthesia, anesthetic and sedative drugs, analgesic and opioid exposure, vasoactive medications, intraoperative blood pressure targets, mean arterial pressure thresholds, definitions and duration of hypotension or hypertension, blood pressure monitoring methods, cerebral oxygenation or perfusion monitoring, co-interventions, follow-up duration, neurocognitive instruments, delirium assessment tools, outcome assessment timing, absolute event rates, mean differences, risk ratios or adjusted estimates when available, losses to follow-up, use of intention-to-treat analysis, funding sources, and conflicts of interest.

For neurocognitive outcomes, numerator and denominator data were extracted whenever reported. When studies only provided p values, graphical data, or incomplete absolute values, this limitation was recorded. Outcomes were grouped according to whether they represented validated delirium or perioperative neurocognitive disorder endpoints, early cognitive or recovery markers, or intermediate hemodynamic and cerebral perfusion variables.

### Risk-of-bias assessment

Risk of bias in included randomized controlled trials was assessed using the Cochrane Risk of Bias 2 tool. The evaluated domains were bias arising from the randomization process, bias due to deviations from intended interventions, bias due to missing outcome data, bias in outcome measurement and bias in selection of the reported result.

Overall risk-of-bias judgments followed the Risk of Bias 2 decision framework. A study was classified as low risk of bias when all domains were judged as low risk. A study was classified as having some concerns when at least one domain raised some concerns but no domain was judged as high risk. A study was classified as high risk of bias when at least one domain was judged as high risk or when multiple domains raised concerns in a way that substantially reduced confidence in the estimated effect.

For trials in which the intervention involved anesthetic or sedative regimens in addition to blood pressure management, potential bias related to deviations from intended interventions, co-interventions, and outcome measurement was specifically considered. Particular attention was given to whether neurocognitive outcome assessors were blinded, whether delirium or cognitive outcomes were prespecified, whether outcome definitions were validated, and whether incomplete outcome data could have influenced effect estimates. Risk of bias was assessed independently by two reviewers, with discrepancies resolved by discussion and, when required, adjudicated by a third reviewer.

### Data synthesis

No meta-analysis was conducted because of substantial clinical and methodological heterogeneity across interventions, populations, surgical settings, anesthetic regimens, blood pressure targets, neurocognitive definitions, assessment instruments, and follow-up timing. A structured narrative synthesis was therefore performed. Results were organized by intervention category and outcome hierarchy, prioritizing postoperative delirium and perioperative neurocognitive disorders, followed by secondary clinical outcomes and intermediate hemodynamic or perfusion-related findings.

Sources of heterogeneity were defined before synthesis. Clinical heterogeneity included age, baseline hypertension, cardiovascular or cerebrovascular comorbidity, baseline cognitive risk, surgical specialty, and procedural complexity. Intervention heterogeneity included absolute versus individualized mean arterial pressure targets, prophylactic versus rescue vasopressor use, ventilation strategies, cerebral oxygenation monitoring, regional or combined anesthesia, and sedative or anesthetic drug selection. Outcome heterogeneity included differences in delirium tools, cognitive tests, hypotension definitions, hypotension duration or burden, follow-up timing, and whether outcomes represented validated neurocognitive endpoints or early recovery markers.

Because of these sources of heterogeneity, results were synthesized narratively according to four intervention categories: blood pressure target or vasopressor-guided strategies, ventilation or cerebral oxygenation strategies, regional or combined anesthetic techniques, and sedative or anesthetic pharmacological regimens. Findings from sedative or anesthetic pharmacological trials were interpreted as indirect or mixed evidence because pharmacological selection may independently influence hemodynamics, pain control, inflammation, emergence, and neurocognitive recovery. Therefore, these trials were not interpreted as evidence that blood pressure management alone reduces perioperative neurocognitive disorders.

Formal publication-bias assessment using funnel plots or statistical tests was not performed because no quantitative synthesis was conducted and the number of studies per outcome was small. No formal certainty-of-evidence assessment was performed because meta-analysis was not feasible. Confidence in the findings was interpreted qualitatively by considering risk of bias, consistency, precision, outcome validity, and directness of evidence.

## Results

### Study selection

**Fig. 1:**
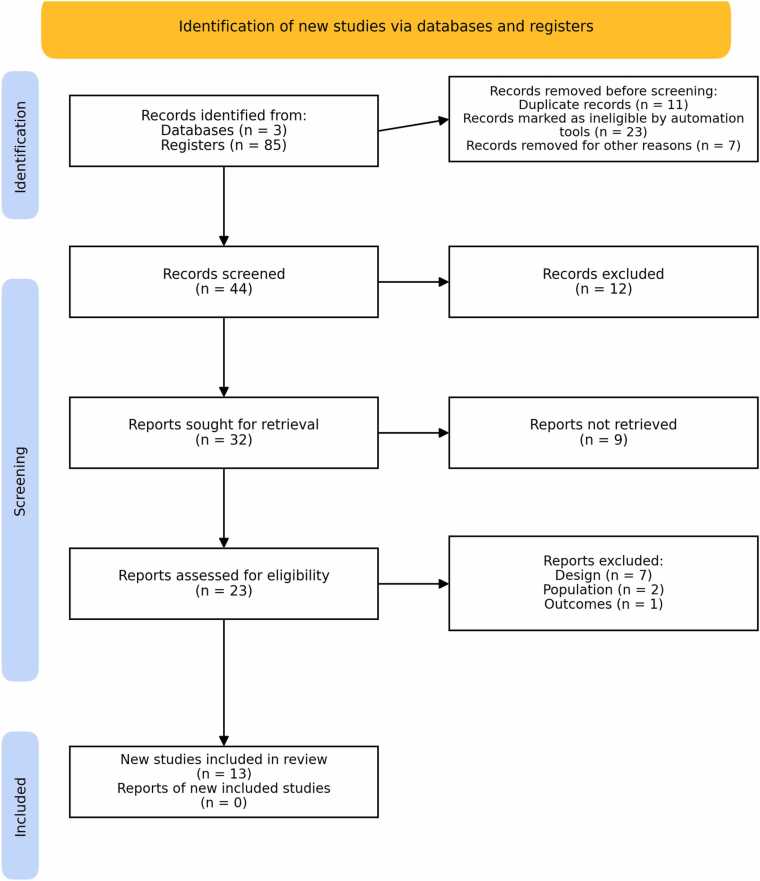
PRISMA flow diagram.

Thirteen randomized controlled trials were included in the review (Fig. 1).

### Study characteristics

Five randomized controlled trials evaluated targeted strategies for intraoperative blood pressure and cerebral/systemic perfusion control (Table 1).^9^, ^10^, ^11^, ^12^, ^13^ Eight randomized controlled trials evaluated anesthetic and sedative strategies compared to placebo or standard care (Table 2).^14^, ^15^, ^16^, ^17^, ^18^, ^19^, ^20^, ^21^

**Table 1:** Targeted strategies for intraoperative blood pressure and cerebral/systemic perfusion control.

**First author, year**	**Population**	**Intervention**	**Comparator**	**Main numerical findings**
Trocheris-Fumery, 2025^9^	Adults > 50 years, ASA ≥II, undergoing major abdominal surgery under general anesthesia. Randomized: 500; analyzed: 473	Prophylactic continuous norepinephrine infusion	On-demand ephedrine boluses	Norepinephrine reduced intraoperative hypotension compared with ephedrine, 15% vs. 74% (*p* < 0.001), and pulmonary complications at 48 h, 17% vs. 31% (*p* < 0.001). Overall 30-day Clavien–Dindo ≥ 1 complications were lower, 44% vs. 58%, mainly due to fewer grade I–II events. No clear differences were found in AKI, cardiovascular, neurological complications, ICU/hospital stay, or 1-month mortality.
Liang, 2021^10^	Elderly patients aged 60–85 years undergoing elective posterior lumbar fusion under general anesthesia. Randomized: 120; 60 per group	Prophylactic norepinephrine infusion	Saline control	Norepinephrine was associated with fewer patients with ≥ 1 in-hospital complication, 18.3% vs. 40% (p = 0.015), and fewer complications at 90 days, 18.3% vs. 43.3% (p = 0.005). Cardiac events were lower, 23.3% vs. 43.3% (p = 0.033), and hospital stay was slightly shorter, 15 vs. 17 days (p = 0.01). Biomarkers suggested less endothelial and cardiac stress, with smaller increases in syndecan−1 and BNP.
Futier, 2017^11^	Adults ≥ 50 years, ASA ≥II, at moderate-to-high risk of postoperative kidney injury, undergoing major surgery under general anesthesia. Randomized: 298; analyzed: 292	Individualized blood pressure management strategy	Standard blood pressure management strategy	Individualized BP management reduced SIRS plus organ dysfunction at day 7, 38.1% vs. 51.7% (adjusted RR 0.73; p = 0.02), renal dysfunction, 32.7% vs. 49.0% (adjusted RR 0.70; p = 0.01), and altered consciousness, 5.4% vs. 15.9% (adjusted RR 0.34; p = 0.007). No significant difference was observed in 30-day mortality, 6.1% vs. 5.5%.
Murphy, 2014^12^	ASA I–III adults undergoing elective shoulder arthroscopy in the beach-chair position under general anesthesia. N = 70	High normocapnia ventilation, E′CO₂ 40–42 mmHg	Mild hypocapnia ventilation, E′CO₂ 30–32 mmHg	High normocapnia reduced cerebral desaturation events, 8.8% vs. 55.6% (*p* < 0.0001; absolute difference −46.7%). MAP, heart rate, and phenylephrine use were similar between groups. Recovery time, pain scores, vomiting, and antiemetic use did not differ significantly.
Yoshimoto, 2005^13^	Adults undergoing posterior lumbar interbody fusion. N = 40; 20 per group	Preoperative epidural block with morphine plus propofol sedation	Inhalational general anesthesia with sevoflurane plus fentanyl	The epidural-propofol strategy produced lower and more stable intraoperative MAP, 57.1 vs. 72.8 mmHg (*p* < 0.001), and reduced the need for continuous antihypertensive infusion. It also reduced postoperative analgesic requirements and PONV. Immediate neurological assessment was possible in 20/20 vs. 15/20 patients, with no serious complications reported.

Abbreviations: AKI: acute kidney injury; ASA: American Society of Anesthesiologists; BNP: B-type natriuretic peptide; BP: blood pressure; E′CO₂: end-tidal carbon dioxide; ICU: intensive care unit; MAP: mean arterial pressure; PONV: postoperative nausea and vomiting; RR: relative risk; SIRS: systemic inflammatory response syndrome. Altered consciousness, cerebral desaturation events, and immediate neurological assessment were interpreted as early neurological or perfusion-related outcomes, not as definitive evidence of sustained perioperative neurocognitive disorder reduction.

**Table 2:** Anesthetic and sedative strategies impacting hemodynamic stability and neurological function versus placebo or standard strategies.

**First author, year**	**Population**	**Intervention**	**Comparator**	**Main numerical findings**
Bi, 2025^14^	Women with thyroid cancer undergoing thyroidectomy under general anesthesia. Randomized: 80; analyzed: 79	IV esketamine during anesthetic induction	Placebo, 0.9% saline	Esketamine reduced the need for continuous vasopressor infusion, 7.5% vs. 28.2% (p = 0.02), and was associated with lower SAS/SDS scores on postoperative day 2 (*p* < 0.05). MAP was higher before intubation and in PACU. Adverse events were similar between groups.
Liu, 2025^15^	Adults with intracranial artery stenosis undergoing elective endovascular treatment under general anesthesia. Analyzed: 95	Remimazolam plus propofol	Propofol alone	The R1 remimazolam-propofol regimen reduced ΔMAP, 21.84 ± 10.77 vs. 26.70 ± 8.49 mmHg, and intraoperative hypotension, 48.4% vs. 79.4% (p = 0.010). Delirium incidence was not significantly different, but CAM-CR severity was lower at day 1 and day 7. PND, MMSE, Barthel Index, and 30-day mortality did not differ.
Qiao, 2023^16^	Patients ≥ 65 years undergoing short elective laser laryngeal surgery under general anesthesia. Randomized: 69; analyzed: 63	Desflurane-based general anesthesia	Propofol-based general anesthesia	MMSE scores did not differ at baseline, PACU, 1 h, 3 h, or 24 h. Cognitive impairment defined as MMSE decrease ≥ 2 points occurred in 9.6% vs. 3.1% (p = 0.583). rSO₂, SpO₂, MAP, HR, recovery time, extubation time, and PACU stay were similar between groups.
Ren, 2019^17^	Patients aged 60–75 years undergoing neuroendovascular procedures under general anesthesia. Randomized: 90; analyzed: 86	High-dose intraoperative IV dexmedetomidine	Lower-dose dexmedetomidine regimens	Higher-dose dexmedetomidine reduced postoperative nimodipine use, remifentanil consumption, and symptomatic cerebral vasospasm, 13.79% vs. 41.38% in the lowest-dose group (p = 0.026). MAP and HR were more stable with higher doses. No differences were observed in GCS, FAS, GOS, cerebral infarction at 30 days, or 3-month neurological outcome.
Zhang, 2019^18^	Patients undergoing elective robot-assisted thoracic surgery under general anesthesia. N = 100; 50 per group	Intraoperative IV dexmedetomidine	Placebo, saline	MAP, HR, and cerebral oxygenation were similar between groups. Dexmedetomidine reduced blood loss, 120 ± 52 vs. 149 ± 67 mL (*p* < 0.05), total propofol dose, 512 ± 347 vs. 723 ± 378 mg (*p* < 0.05), chest tube duration, 3.2 ± 1.8 vs. 4.4 ± 2.7 days (*p* < 0.05), and hospital stay, 4.6 ± 2.2 vs. 6.0 ± 3.1 days (*p* < 0.05). MMSE was higher on POD1 and POD3.
Rajan, 2016^19^	Adults undergoing elective brain tumor resection under balanced general anesthesia. Randomized: 142; analyzed: 139	Intraoperative IV dexmedetomidine	Intraoperative IV remifentanil	Dexmedetomidine produced lower PACU MAP, 88 ± 12 vs. 98 ± 11 mmHg (*p* < 0.001), lower pain scores, 2.9 ± 2.6 vs. 5.1 ± 2.4 (*p* < 0.001), and lower opioid use, 5 vs. 10 mg morphine equivalents (*p* < 0.001). Emergence was slower, but SOMCT and Aldrete scores showed no clinically relevant cognitive recovery difference.
Soliman, 2011^20^	Patients with supratentorial tumors undergoing elective craniotomy under general anesthesia. N = 40	IV dexmedetomidine	Placebo, saline	Dexmedetomidine reduced HR, MAP, ICP, sevoflurane use, and fentanyl consumption, 440 ± 42 vs. 602 ± 73 μg (*p* < 0.001). GCS at 2 h post-extubation was higher, 13.4 ± 1.3 vs. 11.6 ± 1.3 (p = 0.011). Extubation time and antiemetic requirements were also lower in the dexmedetomidine group.
Lauta, 2010^21^	Adults undergoing elective craniotomy for supratentorial lesions. N = 302	Sevoflurane-remifentanil inhalational neuroanesthesia	Propofol-remifentanil total intravenous anesthesia	Time to Aldrete Score ≥ 9 was similar, median 5 min in both groups (p = 0.65). Sevoflurane showed faster eye opening, 6 vs. 8 min (p = 0.014), and extubation, 8 vs. 10 min (p = 0.0018), but more hypotensive episodes per patient, 1.03 vs. 0.61 (p = 0.0167). Brain relaxation and early adverse events were similar.

Abbreviations: AS: Aldrete Score; CAM-CR: delirium severity score based on CAM-CR; FAS: Functional Assessment Scale; GCS: Glasgow Coma Scale; GOS: Glasgow Outcome Scale; HR: heart rate; ICP: intracranial pressure; IV: intravenous; MAP: mean arterial pressure; MMSE: Mini-Mental State Examination; PACU: post-anesthesia care unit; PND: perioperative neurocognitive disorders; POD: postoperative day; rSO₂: regional cerebral oxygen saturation; SAS: Self-Rating Anxiety Scale; SDS: Self-Rating Depression Scale; SOMCT: Short Orientation–Memory–Concentration Test; SpO₂: peripheral oxygen saturation. These trials were interpreted as indirect or mixed evidence because the interventions modified anesthetic or sedative exposure in addition to hemodynamic stability. Early MMSE, GCS, recovery scores, and awakening times were considered early neurological or recovery markers rather than definitive evidence of sustained PND reduction.

### Re-analysis according to intervention type and outcome validity

After reclassification by intervention type and outcome validity, the included trials were interpreted according to the dominant intervention component. Trials evaluating norepinephrine infusion, individualized BP targets, or BP/perfusion-guided strategies were considered direct evidence on intraoperative BP management. Trials evaluating ventilation, epidural anesthesia with propofol sedation, dexmedetomidine, esketamine, remimazolam, desflurane, sevoflurane, or propofol-based regimens were considered indirect or mixed evidence because the intervention modified anesthetic exposure, sedative depth, analgesia, ventilation, or cerebral oxygenation in addition to BP stability.

Therefore, outcome interpretation was restricted according to endpoint validity. Delirium diagnosed with validated instruments and PND/POCD assessed using predefined cognitive criteria were considered primary neurocognitive outcomes. Altered consciousness, early MMSE changes, Glasgow Coma Scale, Aldrete score, awakening time, recovery quality, and cerebral desaturation events were considered secondary, early recovery, or intermediate perfusion outcomes rather than definitive evidence of sustained PND prevention.

### Risk of bias

Risk-of-bias assessment using the Cochrane Risk of Bias 2 tool showed that most included trials were judged to raise some concerns. Twelve of the thirteen trials were classified as having some concerns, mainly because of incomplete reporting or uncertainty in at least one domain, including the randomization process, blinding of outcome assessment, missing outcome data, or selective reporting. One trial was judged as having high overall risk of bias, which introduced greater uncertainty regarding its effect estimates. No trial was classified as low risk of bias across all domains.

**Fig. 2:**
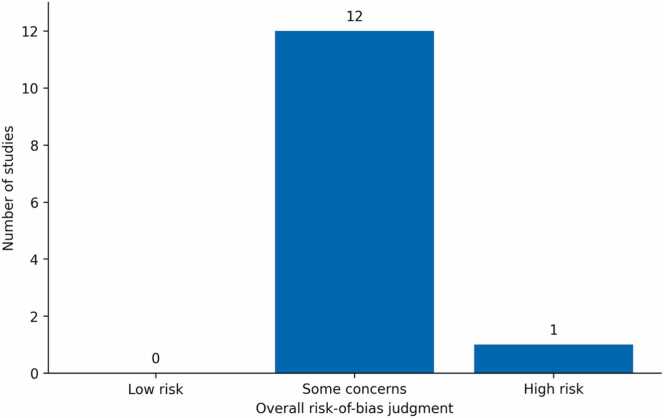
Overall risk-of-bias judgments across included randomized controlled trials.

Overall, the risk-of-bias profile did not suggest widespread serious methodological flaws across the evidence base, but the predominance of some concerns and the presence of one high-risk trial require cautious interpretation of the magnitude of observed effects. This is particularly relevant for neurocognitive outcomes, where outcome definitions, assessment timing, and blinding of outcome assessors were not consistently reported across studies. The overall distribution of risk-of-bias judgments is summarized in Fig. 2.

### Primary outcomes (postoperative delirium and PND/POCD)

In high-risk major abdominal surgery, Futier et al.^11^ found that an individualized blood pressure management strategy reduced the composite outcome of SIRS + organ dysfunction at 7 days (38.1% vs. 51.7%; adjusted RR 0.73; p = 0.02), renal dysfunction (32.7% vs. 49.0%; adjusted RR 0.70; p = 0.01), sepsis and organ dysfunction up to day 30 (adjusted HR 0.66; p = 0.001), and altered consciousness (5.4% vs. 15.9%; adjusted RR 0.34; p = 0.007), with no clear impact on 30-day mortality.

In the setting of controlled ventilation in the beach-chair position, Murphy et al.^12^ showed that high normocapnia (E′CO₂ 40–42 mmHg) maintained stable SctO₂ and significantly reduced the incidence of cerebral desaturation events (CDE) compared to standard mild hypocapnia (≥1 CDE: 8.8% vs. 55.6%; *p* < 0.0001), without significant differences in MAP, vasopressor use, anesthetic recovery, or most immediate clinical outcomes. Lastly, in patients undergoing PLIF, Yoshimoto et al.^13^ found that epidural anesthesia with propofol sedation, compared to inhaled general anesthesia, resulted in lower but stable hypotension (mean MAP 57 vs. 73 mmHg; p < 0.001), less need for nitroglycerin/nicardipine, better pain control, reduced requirement for analgesics and rescue pentazocine, fewer postoperative nausea and vomiting (PONV) episodes, and immediate neurological assessment capability in all patients in the epidural group, with no increase in serious complications.

Taken together, these active strategies for blood pressure control, ventilation, and anesthetic technique suggest that targeted hemodynamic control may be associated with a lower burden of cardiorespiratory, renal, and cerebral perfusion-related complications, as well as less altered consciousness and fewer cerebral desaturation events. However, these outcomes should be interpreted as early clinical or perfusion-related signals rather than definitive evidence of sustained PND reduction (Table 1).

In eight randomized controlled trials evaluating anesthetic and sedative strategies compared to placebo or standard care (Table 2), the main effects observed were related to hemodynamic stability, vasopressor requirements, and early neurological recovery. In patients undergoing thyroidectomy, IV esketamine during induction (Bi et al.^14^) reduced the need for continuous vasopressor infusion (7.5% vs. 28.2% with placebo) and was associated with lower anxiety and depression scores on day 2, along with a biomarker profile suggestive of neuroprotection (increased mBDNF and 5-HT, decreased IGF−1), without an increase in adverse events.

In endovascular treatment of intracranial stenosis, the remimazolam propofol combination (especially the intermediate R1 dose), compared to propofol alone (Liu et al.^15^), reduced MAP variation and intraoperative hypotension incidence (48.4% vs. 79.4%), decreased total propofol and ephedrine use, and was associated with lower delirium severity (CAM-CR) during the first 7 days, though no differences were found in PND, MMSE, Barthel index, or 30-day mortality.

In laser laryngeal surgery in older adults, Qiao et al.^16^ found that desflurane-based anesthesia compared to propofol showed similar hemodynamic and cerebral oxygenation profiles, with low rates of MMSE-defined cognitive decline in both groups and no differences in recovery times.

Three trials evaluating IV dexmedetomidine showed hemodynamic and analgesic benefits with variable impact on neurological outcomes: In neurovascular endovascular therapy (Ren et al.^17^), the high dose (RD3) reduced nimodipine and remifentanil consumption, improved pain control and patient comfort, and was associated with lower rates of symptomatic vasospasm, more stable MAP and HR, with no worsening in Glasgow Coma Scale (GCS), Frontal Assessment Score (FAS), Glasgow Outcome Scale (GOS), or cerebral infarction at 30 days. In robotic thoracic surgery (Zhang et al.^18^), dexmedetomidine vs. placebo did not affect hemodynamics but reduced bleeding, propofol use, chest tube duration, and hospital stay, and was associated with higher QoR−15 and MMSE scores on POD1–3. In brain tumor resection (Rajan et al.^19^), dexmedetomidine vs. remifentanil achieved lower MAP, reduced pain and opioid requirement in PACU, at the cost of slightly slower clinical recovery, but with no relevant differences in SOMCT, Aldrete, or other cognitive recovery indicators.

### Secondary outcomes and Intermediate hemodynamic and perfusion-related findings

In the EPON trial by Trocheris-Fumery et al.,^9^ continuous prophylactic norepinephrine infusion, compared to on-demand ephedrine boluses, markedly reduced intraoperative hypotension (≥1 episode: 15% vs. 74%; p < 0.001) and pulmonary complications at 48 h (17% vs. 31%; p < 0.001), with lower lactate levels in the Post-Anesthesia Care Unit (PACU), and no differences in acute kidney injury (AKI), major cardiovascular or neurological complications, or 30-day mortality. Similarly, in elderly patients undergoing posterior lumbar fusion, Liang T. et al. (10) found that prophylactic norepinephrine was associated with a lower proportion of patients with ≥ 1 in-hospital complication (18.3% vs. 40%; inverse RR 0.46) and at 90 days (18.3% vs. 43.3%), as well as fewer cardiac events and slightly shorter hospital stays, accompanied by higher mean arterial pressure (MAP), lower heart rate, and smaller increases in syndecan−1 and brain natriuretic peptide (BNP), suggesting hemodynamic and endothelial protection without an increase in adverse events. In supratentorial craniotomy, dexmedetomidine compared to placebo (Soliman et al.^20^) significantly decreased HR, MAP, ICP, and sevoflurane/fentanyl consumption, with higher GCS at 2 h post-extubation and reduced antiemetic needs. Meanwhile, comparison between inhaled neuroanesthesia with sevoflurane-remifentanil and TIVA with propofol–remifentanil (Lauta et al.^21^) showed very similar neurological awakening times, slightly faster emergence with sevoflurane but more hypotensive episodes, with no differences in brain relaxation or early complications.

## Discussion

In this systematic review of 13 randomized controlled trials evaluating targeted strategies for intraoperative blood pressure control, cerebral perfusion, and anesthetic technique, protocolized interventions were generally associated with improved hemodynamic stability and favorable early perioperative outcomes compared with standard care. Several studies^9^, ^10^, ^11^, ^12^, ^13^ suggest that prophylactic vasopressor infusions, individualized blood pressure management, ventilatory adjustment, and the use of regional or combined anesthetic techniques may reduce hypotension burden, selected in-hospital complications, and cerebral perfusion-related events. Complementarily, trials^14^, ^15^, ^16^, ^17^, ^18^, ^19^, ^20^, ^21^ suggest that certain sedative and anesthetic regimens based on esketamine, remimazolam, and especially dexmedetomidine may improve hemodynamic stability, optimize pain control, and support better-quality early neurological recovery. However, their impact on persistent postoperative neurocognitive dysfunction (POCD) remains uncertain, and these findings should be interpreted cautiously because pharmacological selection, anesthetic depth, analgesic exposure, surgical context, and baseline patient risk may act as important confounding factors.

When comparing these findings with prior evidence on intraoperative hypotension and organ outcomes, the results from trials focused on blood pressure and perfusion appear both physiologically plausible and clinically coherent. In the EPON trial, Trocheris-Fumery et al.^9^ showed that continuous prophylactic norepinephrine infusion markedly reduced the proportion of patients experiencing at least one hypotensive episode (from 74% to 15%) and lowered early pulmonary complications, without an increase in acute kidney injury or cardiovascular or neurological events. Similarly, in elderly patients undergoing posterior lumbar fusion, Liang et al.^10^ found that prophylactic norepinephrine nearly halved the risk of in-hospital complications, a benefit sustained through 90 days, with fewer cardiac events, higher mean arterial pressure, lower heart rate, and reduced levels of syndecan−1 and BNP—suggesting both hemodynamic and endothelial protective effects. In high-risk major abdominal surgery, Futier et al.^11^ demonstrated that an individualized blood pressure strategy, tailored to each patient's baseline, reduced the combined endpoint of systemic inflammatory response and organ dysfunction by approximately thirteen percentage points, and also significantly lowered rates of renal injury, sepsis, and organ dysfunction up to day 30, in addition to being associated with a threefold reduction in early altered consciousness.

Murphy et al.^12^ showed that maintaining high normocapnia in the beach chair position drastically reduced the incidence of cerebral desaturation events—from 55% to under 10%—despite similar MAP and vasopressor use. Yoshimoto et al.^13^ showed that combining epidural anesthesia with propofol sedation enabled stable, controlled hypotension, reduced need for antihypertensive drugs, better pain control, and fewer nausea and vomiting events, with the added benefit of allowing immediate neurological assessment, without increasing serious complications. Overall, these results are consistent with prior observational studies and series linking intraoperative hemodynamic stability to reduced organ injury and reinforce the concept that avoiding both deep hypotension and abrupt BP fluctuations is a key determinant of perioperative morbidity.

Regarding sedative and anesthetic strategies, the included trials refine and complement previous evidence on POCD. IV esketamine in thyroidectomy, as studied by Bi et al.^,^^14^ was associated with a notable reduction in vasopressor infusion need, lower anxiety and depression scores on day two, and changes in biomarkers consistent with a neuroprotective effect, without increased adverse events aligning with experimental studies describing neuroprotective and mood-modulating properties of this drug. The remimazolam–propofol combination studied by Liu et al.^15^ in endovascular treatment of intracranial stenosis reduced BP variability and incidence of hypotension compared to propofol alone, decreased total propofol and ephedrine use, and was associated with reduced delirium severity during the first week, although no differences were seen in POCD, global cognitive function, or 30-day mortality.

In elderly patients undergoing laser laryngeal surgery, Qiao et al.^16^ reported similar hemodynamic and cerebral oxygenation profiles between desflurane and propofol, with low rates of cognitive decline in both groups suggesting that, in this context, the choice between agents may be based more on practical considerations than neurocognitive differences. The three dexmedetomidine trials show a relatively consistent pattern: In neuroendovascular procedures, Ren et al.^17^ found that high-dose dexmedetomidine reduced nimodipine and remifentanil use, improved pain control and comfort, and was associated with lower symptomatic vasospasm, without worsening neurological or functional scales at 30 days. In robotic thoracic surgery, Zhang et al.^18^ documented reduced bleeding, lower propofol use, shorter chest tube duration and hospital stay, along with improved recovery quality and higher early cognitive scores. In brain tumor resection, Rajan et al.^19^ reported better analgesia and reduced opioid requirement at the cost of slightly slower clinical recovery, but no difference in objective cognitive recovery.

In supratentorial craniotomy, Soliman et al.^20^ showed that dexmedetomidine reduced heart rate, MAP, intracranial pressure, and inhaled anesthetic and opioid use, with improved early consciousness. Meanwhile, Lauta et al.^21^ found very similar awakening times between sevoflurane–remifentanil and total intravenous anesthesia with propofol–remifentanil, with slightly faster emergence but more hypotension with sevoflurane and no significant differences in brain relaxation or early complications. Altogether, these results suggest that regimens based on esketamine, remimazolam, and especially dexmedetomidine may improve hemodynamic stability and early recovery quality but also confirm what has been observed in other studies: evidence for a sustained effect in preventing persistent POCD remains limited and heterogeneous.

This review has methodological strengths that support the internal validity of its conclusions. It was limited to RCTs comparing targeted interventions to standard care, covering a broad range of surgical contexts including major abdominal, spinal, thoracic, neurosurgical, neuroendovascular, and head and neck procedures. Risk of bias was systematically assessed using the RoB 2 tool, with 12 out of 13 studies rated as having "some concerns" and only one trial deemed high risk. This indicates no major methodological flaws, but also reveals recurring weaknesses in aspects such as randomization procedures, outcome assessment blinding, and complete results reporting—requiring cautious interpretation of effect sizes. Additionally, considerable heterogeneity across patient populations, surgery types, definitions of hypotension and cerebral desaturation, drug combinations, neurocognitive scales, and follow-up durations limited the feasibility of formal meta-analysis and reduced the precision of pooled estimates on POCD.

From a clinical practice standpoint, findings from these trials support a proactive, protocolized approach to hemodynamic and intraoperative sedation management, especially in high-risk patients due to advanced age, cardiovascular comorbidities, or procedural complexity. Results from (9–11) support the use of prophylactic norepinephrine infusions or individualized blood pressure targets in selected contexts to reduce the burden of hypotension and organ complications without increasing adverse events. Studies^12^, ^13^ highlight the importance of integrating ventilation control and rational use of regional or combined techniques to optimize cerebral perfusion and facilitate early neurological assessment.

In terms of sedative and anesthetic agents, findings^14^, ^15^, ^17^, ^18^, ^19^, ^20^ suggest that incorporating esketamine, remimazolam, or dexmedetomidine into standard regimens may improve hemodynamic stability, reduce anesthetic or opioid requirements, and enhance early recovery quality—but these do not yet justify their adoption as specific long-term POCD prevention strategies. Larger trials with longer follow-up are needed to standardize definitions of delirium and perioperative neurocognitive disorders, employ comprehensive neuropsychological batteries, and directly compare hemodynamic and sedative algorithms in key subgroups such as frail patients, those with chronic hypertension, or those with baseline cognitive impairment.

An additional source of heterogeneity was the long publication period covered by the included trials. Studies published between 2005 and 2025 differed substantially in the terminology, diagnostic criteria, and tools used to evaluate postoperative cognitive outcomes. Earlier studies frequently used terms such as POCD, altered consciousness, early cognitive decline, awakening quality, or isolated cognitive test changes, whereas more recent perioperative literature increasingly uses the broader concept of PND and places greater emphasis on validated delirium tools, standardized cognitive testing, baseline cognitive status, and longer follow-up. This temporal variability limited direct comparability across trials and was one of the main reasons for avoiding quantitative pooling of neurocognitive outcomes.

## Limitations

This review has several limitations. First, substantial clinical and methodological heterogeneity was present across studies, including differences in surgical specialties, baseline patient risk, age distribution, hypertension status, anesthetic technique, sedative and analgesic exposure, BP targets, definitions of hypotension, monitoring methods, and follow-up duration. Second, neurocognitive outcomes were inconsistently defined. Some studies assessed validated delirium or PND outcomes, whereas others reported altered consciousness, early MMSE changes, awakening scores, cerebral desaturation events, or recovery-quality measures, which cannot be considered equivalent to sustained PND.

Third, several trials reported statistical significance without complete numerator/denominator data, absolute event rates, mean differences, or precision estimates for all relevant neurocognitive outcomes, limiting comparability and precluding meta-analysis. Fourth, anesthetic and sedative co-interventions introduced potential confounding, particularly in trials where the intervention modified both BP stability and pharmacological exposure. Fifth, the long time span of included studies introduced temporal heterogeneity because definitions and tools for POCD/PND changed over time. Finally, subgroup analyses by patient risk, including elderly patients, chronic hypertension, frailty, or baseline cognitive impairment, were limited by incomplete reporting and small numbers of trials within each subgroup.

## Conclusions

Protocolized intraoperative BP and perfusion-related strategies may improve hemodynamic stability and reduce selected early perioperative complications, particularly hypotension burden and some early markers of cerebral perfusion or neurological recovery. However, current randomized evidence remains insufficient to conclude that these strategies produce a sustained reduction in postoperative delirium or long-term PND. The interpretation is limited by heterogeneity in BP targets, anesthetic and sedative co-interventions, surgical populations, neurocognitive definitions, assessment tools, and follow-up duration.

Therefore, intraoperative management should prioritize avoidance of severe or prolonged hypotension and marked BP fluctuations, preferably through individualized and protocolized approaches in high-risk patients. Nevertheless, BP control should be considered one component of a broader multimodal strategy rather than an isolated intervention proven to prevent PND. Future trials should use standardized delirium and PND definitions, report absolute event rates and effect estimates, stratify patients by baseline neurocognitive and cardiovascular risk, and include longer follow-up to determine whether improved intraoperative hemodynamic stability translates into meaningful neurocognitive benefit.

## CRediT authorship contribution statement

**Paula Henríquez Miranda:** Conceptualization, Study selection, Data curation, Writing – original draft. **Daniela Peña Pérez:** Study selection, Data extraction, Writing – original draft. **Juan Pablo Maestre Aguancha:** Methodology, Validation, Writing – review & editing. **Mariela Alejandra Cabas Ramos:** Formal analysis, Writing – review & editing. **Juan Sebastián Cuellar Cadena:** Formal analysi, Writing – review & editing. **Francisco Javier Gómez Ballesta:** Conceptualization, Methodology, Supervision, Writing – review & editing. **Juan Pablo Alzate Granados:** Methodology, Data curation, Writing – review & editing. All authors reviewed and approved the final version of the manuscript.

## Consent for publication

Not applicable.

## Ethical statement

This study is a systematic review based exclusively on previously published data and did not involve direct contact with human participants, identifiable personal data, or animal experimentation. Therefore, ethics committee approval and informed consent were not required.

## Funding

The authors declare that this systematic review did not receive specific funding from public agencies, academic institutions, non-profit organizations, or industry.

## Declaration of competing interest

The authors declare that they have no known competing financial interests or personal relationships that could have appeared to influence the work reported in this paper.

## Data Availability

All data cited in this review are publicly available through the original publications listed in the references. Source data for figures are provided with this paper.

## Footnotes

Appendix A Supplementary data associated with this article can be found in the online version at doi:10.1016/j.jatmed.2026.06.002.
