# Supplementary material for: Intraoperative blood pressure strategies and neurocognitive outcomes: A systematic review
Source: J Anesth Transl Med. 2026 Jun 26;5(2):124–33. doi: 10.1016/j.jatmed.2026.06.002 (PMC13319371; doi:10.1016/j.jatmed.2026.06.002)
Supplement: Supplementary file 1 — Supplementary material [file mmc1.pdf]

PubMed:

(("Anesthesia, General"[MeSH] OR "general anesthesia"[tiab] OR "under general anesthesia"[tiab]) AND ("Intraoperative Care"[MeSH] OR intraoperative[tiab] OR "during surgery"[tiab]) AND ("Blood Pressure"[MeSH] OR "Hypotension, Controlled"[MeSH] OR "Hemodynamics"[MeSH] OR "Monitoring, Physiologic"[MeSH] OR "hemodynamic monitoring"[tiab] OR "goal-directed therap*"[tiab] OR "goal directed therap*"[tiab] OR "blood pressure management"[tiab] OR "blood pressure control"[tiab] OR "mean arterial pressure"[tiab] OR MAP[tiab] OR vasopressor*[tiab] OR vasodilator*[tiab] OR vasoactive[tiab] OR "vasoactive agent*"[tiab]) AND ("Delirium"[MeSH] OR "postoperative delirium"[tiab] OR "Postoperative Complications"[MeSH] OR "Cognition Disorders"[MeSH] OR "Neurocognitive Disorders"[MeSH] OR "postoperative cognitive dysfunction"[tiab] OR POCD[tiab] OR "cognitive decline"[tiab] OR "cognitive impairment"[tiab] OR neurocognit*[tiab] OR ("Mortality"[MeSH] OR mortalit*[tiab]) AND (neurolog*[tiab] OR brain[tiab])) AND ("Adult"[MeSH] OR adult*[tiab])) NOT ("Child"[MeSH] OR "Infant"[MeSH] OR "Adolescent"[MeSH])

Embase:

(('general anesthesia'/exp OR 'general anesthesia':ti,ab OR 'under general anesthesia':ti,ab) AND ('intraoperative care'/exp OR intraoperative:ti,ab OR 'during surgery':ti,ab) AND ('blood pressure'/exp OR 'controlled hypotension'/exp OR 'hemodynamics'/exp OR 'hemodynamic monitoring'/exp OR 'physiologic monitoring'/exp OR 'goal directed therapy':ti,ab OR 'goal-directed therapy':ti,ab OR 'blood pressure management':ti,ab OR 'blood pressure control':ti,ab OR 'mean arterial pressure':ti,ab OR MAP:ti,ab OR vasopressor*:ti,ab OR vasodilator*:ti,ab OR vasoactive:ti,ab OR 'vasoactive agent*':ti,ab) AND ('delirium'/exp OR 'postoperative delirium'/exp OR 'cognitive defect'/exp OR 'neurocognitive disorder'/exp OR 'postoperative cognitive dysfunction'/exp OR 'postoperative cognitive dysfunction':ti,ab OR POCD:ti,ab OR 'cognitive decline':ti,ab OR 'cognitive impairment':ti,ab OR neurocognit*:ti,ab OR (mortality:ti,ab OR 'mortality'/exp) AND (neurolog*:ti,ab OR brain:ti,ab)) AND ('adult'/exp OR adult*:ti,ab) AND [humans]/lim) NOT ('child'/exp OR 'infant'/exp OR 'adolescent'/exp)

Lilacs:

((mh:"Anesthesia, General" OR tw:("general anesthesia" OR "under general anesthesia")) AND (mh:"Intraoperative Care" OR tw:(intraoperative OR "during surgery")) AND (mh:"Blood Pressure" OR mh:"Hypotension, Controlled" OR mh:"Hemodynamics" OR mh:"Monitoring, Physiologic" OR tw:("hemodynamic monitoring" OR "goal-directed therap*" OR "goal directed therap*" OR "blood pressure management" OR "blood pressure control" OR "mean arterial pressure" OR MAP OR vasopressor* OR vasodilator* OR vasoactive OR "vasoactive agent*")) AND (mh:"Delirium" OR tw:"postoperative delirium" OR mh:"Cognition Disorders" OR mh:"Neurocognitive Disorders" OR tw:("postoperative cognitive dysfunction" OR POCD OR "cognitive decline" OR "cognitive impairment" OR neurocognit*) OR (mh:"Mortality" OR tw:mortalit*) AND tw:(neurolog* OR brain)) AND (mh:"Adult" OR tw:adult*)) NOT (mh:"Child" OR mh:"Infant" OR mh:"Adolescent")
